# Designing a modular digital health curriculum for pharmacy education: a stakeholder-driven expert workshop study

**DOI:** 10.3389/fpubh.2026.1888486

**Published:** 2026-07-29

**Authors:** Mélanie Raimundo Maia, João Gregório

**Affiliations:** 1 Global Health and Tropical Medicine (GHTM), LA-REAL, Instituto de Higiene e Medicina Tropical (IHMT), Universidade NOVA de Lisboa, Lisboa, Portugal; 2 WHO Collaborating Centre on Health Workforce Policies and Planning, Universidade NOVA de Lisboa, Lisboa, Portugal; 3 CBIOS, ECTS, Universidade Lusófona, Lisboa, Portugal

**Keywords:** artificial intelligence, co-design, competency-based education, DECODE framework, digital health, digital public health, instructional design, pharmacy education

## Abstract

Digital health technologies are increasingly transforming pharmaceutical care, yet pharmacy education has been slow to integrate structured digital health content into curricula. This study aimed to co-design a modular digital health curriculum framework for pharmacy education through a qualitative, stakeholder-driven process grounded in the ADDIE instructional design model and informed by the DECODE digital health competency framework. Two structured expert workshops, conducted at the 10th PCNE Working Symposium (Innsbruck, Austria, February 2026), engaged 30 participants from 13 European countries in three sequential co-design activities: competency needs mapping, curricular design sprints, and framework integration. Competency prioritization identified four workshop-level agreement pillars: artificial intelligence in healthcare, electronic health records, ethics and legal–professional foundations, and digital health literacy, alongside strong support for telehealth and wearable/sensor technologies. Eight module outlines were co-designed, spanning electronic health records, health applications, large language models, telehealth, digital health literacy, artificial intelligence in healthcare and ethics/legal/regulatory topics, and were organized into a three-tier curricular sequence (foundational, applied and advanced) that differentiates mandatory core, pathway-specific and postgraduate/CPD content. Reported feasibility was shaped by faculty expertise, curriculum crowding, governance, accreditation and institutional digital maturity, while accreditation mandates, European regulatory directives, interprofessional collaboration and pilot programs emerged as key enablers. Participants critically appraised the DECODE framework, noting its medical education origins and advocating action-oriented learning outcomes and pharmacy-specific contextualization rather than direct transposition. By combining Theory of Change with an instructional, competency-based approach, this study provides an early-stage multinational co-designed digital health curriculum blueprint, tailored for pharmacy education, representing the Analysis and Design phases of ADDIE; Development, Implementation and Evaluation phases constitute future work. It offers an implementation-conscious blueprint for strengthening the digital readiness of the pharmacy workforce.

## 1 Introduction

Digital health (DH) technologies - including mobile applications, wearable devices, telepharmacy platforms, and artificial intelligence (AI) tools - are fundamentally transforming pharmaceutical care and reshaping the pharmacist’s role in health systems worldwide (1). As healthcare delivery becomes increasingly digitized, pharmacists are expected to navigate electronic health records (EHRs), evaluate digital therapeutics, support patients in using health applications and wearable sensors, and integrate AI-assisted decision support into clinical workflows. The pharmacist’s evolving role in digital health ecosystems - encompassing electronic prescribing, telepharmacy, clinical decision support, and digital therapeutics - has been documented across diverse healthcare systems (2–4). The adoption of e-prescription systems, for instance, has been shown to enhance workflow efficiency, medication safety, and patient satisfaction, though implementation barriers related to system interoperability and organizational readiness persist (3). These technological shifts demand a workforce equipped not only with traditional pharmaceutical knowledge but also with the digital competencies necessary to deliver safe, effective, and patient-centered care in digitally enabled environments.

Despite this transformation, pharmacy education has been slow to respond. The 2021 International Pharmaceutical Federation (FIP) report on Digital health in pharmacy education documented significant gaps in how pharmacy curricula address digital health topics (1). The global survey underlying the report revealed that clinical reasoning and evidence-based digital medicine were the least covered digital health concepts, with only 24 and 26% of programs reporting coverage, respectively. Cyber security (29%), entrepreneurship (31%), and patient empowerment (32%) were similarly under-represented. The report also highlighted a persistent lack of faculty expertise, confusion between digital health education and online education delivery, and the absence of systematic frameworks for integrating digital competencies into pharmacy curricula. Early exposure to digital competencies was identified as a key enabler of professional adoption, yet structured approaches to achieving this remain rare (1, 5).

Recognizing the urgency of this gap, FIP Development Goal 20 calls for a digitally literate pharmaceutical workforce capable of leveraging technology to improve health outcomes. However, translating this aspiration into concrete curricular structures requires competency frameworks, pedagogical models, and stakeholder engagement processes that are currently underdeveloped in the pharmacy domain. The DECODE - Digital Health Competencies in Medical Education - framework has emerged as a comprehensive reference, encompassing four domains, 19 competencies, and 178 learning outcomes (33 mandatory and 145 discretionary) derived through an international Delphi consensus process (6). Despite aiming the medical education, DECODE was developed within a strong multidisciplinary and interprofessional context, involving 211 international experts from 79 countries with diverse professional backgrounds in clinical medicine, health informatics, digital health research, and public health policy. This interdisciplinary foundation suggests this framework can be applied to other digital health contexts beyond its primary clinical medical focus; indeed, its inclusion criteria explicitly incorporated competencies formulated for all health workers to improve generalizability. The interprofessional nature of DECODE’s development provides a robust, cross-cutting blueprint for establishing digital health competencies across the wider health workforce, namely within the pharmacy education context.

Evidence from systematic reviews supports the effectiveness of well-designed digital and technology-enhanced learning approaches in health professions education. E-learning programs have demonstrated capacity to build healthcare professional competencies when designed with clear learning objectives and interactive elements (7). Adaptive e-learning systems have shown promise in personalizing instruction for health professionals and students (8). Gamification strategies - including challenges, badges, leaderboards, and social elements - can increase engagement when aligned with pedagogical objectives, though effects on learning outcomes remain variable (9, 10). The growing integration of AI tools in pharmacy education has been the subject of recent scoping reviews, which document both expanding applications - including personalized learning, AI-assisted assessment, and academic integrity challenges - and persistent gaps in structured curricular integration and faculty preparedness (11) These findings suggest that a modular digital health curriculum for pharmacy education could benefit from blended, technology-enhanced pedagogical approaches.

Beyond pedagogical delivery methods, effective digital health curriculum development requires grounding in competency-based education (CBE) principles and implementation science. CBE frameworks emphasize the alignment of learning outcomes, instructional activities, and assessment around defined professional competencies, ensuring that graduates meet explicit performance standards (12). Implementation science further highlights that curriculum adoption depends not only on content validity but on contextual fit, stakeholder buy-in, faculty capacity, and organizational readiness - factors directly addressed in this study’s design.

Despite growing recognition of the need for digital health education in pharmacy, no published study reports a systematically co-designed, competency-based digital health curriculum framework specifically developed for and with pharmacy stakeholders. The pharmacy profession has been embracing the development of competency frameworks, educational outcomes, and trustable professional activities (12), but has still lacked meaningful integration of CBE approaches into digital health education. Existing efforts tend to be institution-specific, address individual technologies in isolation, or lack grounding in validated, pharmacy-relevant competency frameworks - a gap independently noted by FIP (13) and confirmed by recent scoping reviews of digital health technologies in pharmacy education (14). The fragmentation of digital public health training across European programs has been documented more broadly (15, 16) underscoring the need for structured, multi-stakeholder curriculum development processes.

This study aimed to design a modular digital health curriculum framework for pharmacy education using a qualitative, stakeholder-driven ADDIE - Analysis, Design, Development, Implementation, Evaluation - process (17). Specifically, we sought to: (a) identify and prioritize digital health competencies for pharmacy students by applying the DECODE framework; (b) translate prioritized competencies into modular curriculum components with defined learning outcomes, teaching methods, and assessment strategies; and (c) elicit expert perspectives on barriers, enablers, and contextual factors affecting curriculum implementation across diverse European pharmacy education settings.

## 2 Methods

Epistemologically, the present study is grounded in a constructivist orientation, assuming that knowledge about curriculum needs and implementation conditions is co-constructed through interaction among stakeholders embedded in specific professional and institutional contexts (29). The methodological tradition is best characterized as participatory co-design, combining elements of framework analysis (applying the DECODE and FIP frameworks as deductive sensitizing structures) and reflexive thematic analysis (generating inductive themes from feasibility and implementation data) (30, 31). This approach aligns with a pragmatic, action-oriented research tradition in health professions education, oriented toward producing contextually grounded and practically actionable outputs.

### 2.1 Study design

This study was a qualitative, co-design study using structured expert workshops as focus-group-style data collection events. The study was embedded in the Analysis and Design phases of the ADDIE instructional design model (17), a five-phase systematic framework (Analysis, Design, Development, Implementation, Evaluation) widely used in health professions education to structure curriculum and training design (18–20). The study was aligned with Theory of Change (ToC) principles (21) operating under the assumption that engaging key stakeholders in structured co-design processes, grounded in established digital health competency frameworks, would lead to context-relevant and pedagogically robust learning modules more likely to be adopted and sustained. The study was registered on the Open Science Framework repository.^1^

Workshops facilitators (MM and JG) are both health systems senior researchers with active professional interest in digital health, working in different institutions and with different backgrounds. MM is a senior researcher, expert in Global Health, Digital Health and Health Workforce policy and planning. JG is a senior academic leader whose expertise spans Global Health, Pharmacy services, Health services research, Digital Health and Implementation science. To mitigate potential influence, facilitation followed a structured protocol with pre-defined activities, materials, and timing, minimizing facilitator-directed discussion. During plenary sessions, facilitators served primarily as moderators and theme clusterers rather than contributors. No close relationship existed between the research team and the participants, as recruitment was open to all conference attendees. The analytic process included explicit reflexive review: both researchers maintained analytic memos during coding and discussed assumptions that might could have influenced theme selection. Final themes were grounded in patterns supported across multiple independent groups rather than isolated facilitator-salient comments.

### 2.2 Participants and setting

Two three-hour workshops were held on February 17 and 18, 2026, at the Ágnes-Heller-Haus, Innsbruck, Austria, as part of the 10th Pharmaceutical Care Network Europe (PCNE) Working Symposium. A total of 30 participants provided informed consent and took part in the workshops (*n* = 14 in Workshop 1, *n* = 16 in Workshop 2). Recruitment was conducted through open registration at the 10th PCNE Working Symposium. All conference attendees were eligible to register for the workshops; no formal exclusion criteria were applied beyond conference attendance. As the PCNE Symposium primarily attracts individuals with expertise in pharmaceutical care, pharmacy education, digital health, and health systems research, the sample is purposive by virtue of conference selection and self-registration. The total number of eligible conference attendees was not formally recorded, and the participation rate therefore cannot be precisely calculated. Self-selection into the workshops means that participants were likely to be more innovation-oriented and engaged with digital health topics than the broader pharmacy education community. The sample was predominantly academic and European, consistent with the PCNE membership profile. These characteristics are acknowledged as limitations and discussed further in the Strengths and Limitations section.

Participant characteristics are summarized in Table 1. Reported aggregated variables include professional group, current occupation, and represented countries of origin. In accordance with GDPR data minimization and integrity and confidentiality principles [GDPR (EU) 2016/679; (22)] further individual-level variables, including gender and years of experience, were not collected, to prevent deductive disclosure within this small, identifiable professional community.

**Table 1:** Participant characteristics (*N* = 30).

**Characteristic**	***n***	**%**
Professional group		
Pharmacist (Academia)	18	60.0
Pharmacist (Community)	4	13.3
Clinical Pharmacist	4	13.3
Health Systems Researcher	3	10.0
Clinical Toxicologist	1	3.3
Occupation		
Professor	16	53.3
PhD Student	4	13.3
Post-doctoral Researcher	3	10.0
Researcher	2	6.7
Director/Department Head	3	10.0
Dean	1	3.3
Other	1	3.3
Countries represented	13	—
Workshop allocation		
Workshop 1	14	46.7
Workshop 2	16	53.3

Countries: Austria, Belgium, Denmark, Germany, Ireland, Kosovo, Netherlands, Norway, Portugal, Serbia, Spain, Sweden, Switzerland.

### 2.3 Workshop protocol

Each workshop was structured around a single overarching research question: “What digital health competencies should be prioritized in pharmacy education, and how can they be translated into modular curriculum components and learning experiences that are feasible and relevant across pharmacy practice settings?” Participants worked through three sequential, interactive activities within each three-hour session, organized as follows:

Activity 1–competency needs mapping (30 min). Participants were divided into small groups of 4–6 and provided with a competencies pool worksheet (Supplementary material) adapted from the DECODE framework (6) and aligned with FIP digital health concepts. Each group was tasked with reviewing the competencies, discussing their relevance to pharmacy education, and identifying the 5 most critical core digital health competencies for pharmacy students in their context. Each selected competency was recorded on a post-it note and a brief rationale.

This activity was followed by Plenary Synthesis (30 minutes). Each group presented their top competencies (about 4 minutes per group). Facilitators clustered the presented competencies thematically according to DECODE domains. This was followed by an open discussion to explore convergences, divergences, and contextual explanations, resulting in a prioritized and consensual list based on the number of groups selecting each competency.

Activity 2–curricular design sprint (40 min). Working in the same groups, participants selected up to two of their priority competency clusters and draft one short module outline. For each module, groups specified: (a) a title and target learners; (b) up to three main learning outcomes aligned with Bloom’s taxonomy and FIP digital health survey concepts; (c) suggested teaching and learning methods informed by evidence on digital education and gamification in health professions (7–9); and (d) one or more example assessment approaches.

Activity 3–framework integration (35 min). Groups presented their module outlines, while overlaps and gaps across proposals were identified and feasibility issues-including time, resources, integration into existing curricula, and faculty capacity-were discussed. When applicable, modules were reorganized into a tentative sequence (foundational to advanced) reflecting how they might be implemented in participants’ own contexts.

Four groups were formed in each workshop, yielding eight groups in total across both sessions. Each group worked independently on competency prioritization (Activity 1), module design (Activity 2), and framework integration (Activity 3). All plenary discussions and group presentations were audio recorded and transcribed (verbatim). Materials provided to participants included the DECODE competencies pool worksheet, paper, pens and post-it blocks for taking notes.

To promote equitable participation and manage group dynamics, facilitators employed structured turn-taking during group presentations, used open-invitation prompts to elicit contributions from quieter participants, and explicitly invited dissenting or alternative views during plenary discussion. Group size (4–6 participants) was kept small to facilitate broad engagement. Where one or two voices appeared dominant in plenary discussion, facilitators checked for alternative perspectives. Both workshops followed the same protocol, materials, and facilitation approach to ensure consistency across sessions. Post-workshop, facilitators wrote brief field notes documenting any notable group dynamics, which were used as contextual information during data analysis.

### 2.4 Theoretical and conceptual framework

The study drew on four complementary theoretical and conceptual frameworks. First, the ADDIE instructional design model (17) provided the overarching structure, with the workshops corresponding to the Analysis phase (identifying needs, priorities, and implementation conditions) and the Design phase (translating needs into modular curriculum components). Second, the DECODE framework (6) served as the primary competency reference, offering a validated taxonomy of digital health competencies organized into four domains: (1) Professionalism, ethics, law and regulation in digital health; (2) Digital health technologies and applications; (3) Data, information management and records; and (4) Advanced digital health applications. For the purposes of the workshop, these domains were used as a sensitizing scaffold rather than as a fixed curriculum template. Third, the FIP global study on digital health in pharmacy education (1) provided a pharmacy-specific benchmark on curricular gaps, readiness, and reported barriers to digital health teaching. Fourth, the WHO Theory of Change guidance (21) informed the project’s logic model, linking stakeholder engagement and design inputs to expected intermediate outcomes (co-designed curriculum proposals, implementation insights, educator-oriented design recommendations) and longer-term impact (digitally competent pharmacy workforce, improved pharmaceutical care).

### 2.5 Data analysis

Data analysis was conducted by the two authors (MM and JG). Transcripts from all workshop sessions were first independently reviewed by both researchers in a familiarization phase, involving repeated reading and preliminary annotation. Deductive coding was then applied by MM against the DECODE competency domains and the FIP digital health concepts, using a structured coding matrix developed *a priori* as the primary codebook scaffold (Supplementary material). This was followed by an inductive phase in which both researchers reviewed the transcript data for emergent themes not captured by the deductive framework, with particular attention to feasibility-related content. AI-based tools (NotebookLM, Gemini 3 - Google Labs; ChatGPT 5.3 - OpenAI) were used in an assistive capacity during the initial familiarization and preliminary coding phase only; all coding decisions, theme definitions, and interpretive synthesis were subsequently reviewed, revised, and validated by both researchers. Conflict in coding were initially resolved through iterative discussion until an agreement was reached. If agreement could not be reached, a third, neutral researcher would act as an arbitrator. No formal intercoder reliability coefficient was calculated, consistent with the interpretive, qualitative epistemology of the study (30). No qualitative data analysis software was used; analysis was conducted directly on annotated transcripts and coding matrices.

Given the structured, workshop-based design and the use of a pre-defined competency framework as a deductive scaffold, thematic saturation in the inductive sense was not the primary criterion for closing data collection; rather, the analysis aimed to cover all competency domains and all group outputs comprehensively. Convergence across groups was used as a proxy indicator that the major themes had been adequately captured.

The analytic process followed a hybrid inductive-deductive design (23, 24), as follows:

Stage I—Deductive mapping against DECODE/FIP: In this first deductive round, all participant statements were mapped against the four DECODE domains and the FIP digital health competency concepts as sensitizing categories (e.g., A group statement such as “students need to know how to use EHRs for medication review,” coded to DECODE Domain 3/Competency 3.3 (Data, Information Management and Records-Electronic Health Records) and to FIP concept: Clinical reasoning/Health information systems.)

Stage II—Structured coding matrix: This step produced a coding matrix of competency references, bundling proposals, and adaptation rationales across all eight groups of participants (e.g., Matrix with Competencies 4.2 (AI in healthcare) and 3.3 (EHRs) referenced by all 8 groups, while Competency 3.1 (data governance) referenced only in the context of deferral to Continuing Professional Development, CPD).

Stage III—Inductive pass on uncaptured segments: At this stage, data segments not captured by the DECODE/FIP scaffold - particularly those relating to implementation conditions, pedagogical preferences, assessment challenges, and AI-specific disruptions - were openly coded for emerging patterns (e.g., a statement such as “oral exams are causing student anxiety because of AI” does not map to any DECODE competency. It was openly coded as “Assessment disruption” and “Student anxiety and acceptability concerns“, contributing to the inductive Theme 10 - AI as pedagogical disruptor).

Stage IV—Examining convergence: Candidate themes were generated by collating codes across both passes and examining convergence across multiple groups and workshops (e.g., the code “bundling of digital diagnostics, wearables, and health apps” appeared independently in 6 of 8 groups, producing similar bundling proposals; since it met the convergence criterion (the minimum of having at least three groups elaborating on it), and the pattern was robust enough to qualify as a cross-cutting theme, it was retained as supporting Theme 4 - Patient-facing digital technologies as integrated clusters).

Stage V—Iterative theme naming: Themes were refined, reviewed against the full dataset, and named iteratively. (e.g., an early candidate theme labelled “practical barriers to implementation” was refined through review to become Theme 9 - Feasibility is shaped by barriers, enablers, and contextual factors, clearly distinguishing the three-part structure revealed in the data).

Themes were retained if supported by data from at least three independent group outputs (i.e., at least three of the eight small groups produced data converging in the same direction). This convergence criterion was used as a proxy for analytical robustness. Themes derived primarily from the deductive coding stage are distinguished from those that emerged predominantly from the inductive feasibility analysis.

Together, the materials of support - the workshop protocol with pre-registration, competency pool worksheet (Supplementary material), and Tables of Results, including the coding matrix are intended to provide sufficient documentation to allow replication or adaptation of the co-design methodology in other health education contexts

### 2.6 Ethical considerations

The study was approved by the Ethics Committee of the School of Sciences and Health Technologies, Universidade Lusófona, under approval number CE. ECTS/P17-25. All participants provided written informed consent prior to participation, including explicit permission for audio recording of workshop sessions. Data were pseudonymized during processing, then irreversibly anonymized prior to storage, in accordance with institutional policies and the General Data Protection Regulation (GDPR, EU 2016/679) - complying with the principles addressing the risk of re-identification, including storage limitation, data minimization, and integrity and confidentiality (22). Access to identifiable data was restricted to the research team and only for the time required to transcribe, verify, and anonymize the recordings. Anonymized data will be retained for 5 years and then securely deleted. No sensitive personal data (for example, health records or financial information) were collected. Publications report consensus-based and thematic findings rather than attributing comments to identifiable individuals. Participants retained the right to request access to, rectification of, or erasure of any personal data relating to them, within the limits that apply once data have been anonymized. Because AI-based tools were used in an assistive role during the initial analytic phase, particular care was taken to ensure this use was consistent with the study’s data protection requirements. Prior to any input into AI tools, transcripts were irreversibly anonymized: any directly or indirectly identifying information were removed from the text. No raw or pseudonymized transcripts were entered into third-party AI platforms. AI tools were used exclusively with anonymized text fragments for the purposes of preliminary familiarization and pattern detection. All final coding decisions, theme definitions, and interpretive synthesis were conducted, reviewed, and validated by the research team.

## 3 Results

The participant panel represented diverse professional backgrounds and institutional contexts across Europe. Professional groups included academic pharmacists (the largest group), community pharmacists, clinical pharmacists, health systems researchers, and clinical toxicologists. Occupational roles ranged from professors, who constituted the majority, to PhD students, post-doctoral researchers, department heads, directors, and a dean. Participants were affiliated with institutions across 13 European countries: Austria, Belgium, Denmark, Germany, Ireland, Kosovo, the Netherlands, Norway, Portugal, Serbia, Spain, Sweden, and Switzerland. Table 1 presents participant characteristics in detail.

### 3.1 Thematic coding framework

The workshop transcripts were first deductively coded against the DECODE competency structure and the FIP digital health concepts and reported gaps, and then further analyzed using an inductive-deductive approach focused on implementation feasibility. Across the four transcripts, 10 cross-cutting themes were identified. These themes provided the analytical framework for interpreting the competency prioritization, module design, curricular sequencing, and implementation discussions.

The 10 final themes (Table 2) represent patterns that were consistent across multiple groups and both workshops, with each theme supported by data from at least three independent group outputs, meaning at least three of these eight groups produced data pointing in the same direction (a convergence criterion used as a proxy for analytical robustness). Themes 1–7 derived primarily from the deductive stage; Themes 8–10 emerged predominantly from the inductive feasibility analysis.

**Table 2:** Overview of the thematic coding framework.

Theme	Primary analytic focus	Main DECODE anchors	Main implementation relevance
1. Foundational digital professionalism is non-negotiable*	Core foundations	1.1, 1.2	Safe, ethical, and secure baseline
2. Digital health literacy is a transversal competence*	Communication, equity, patient support	2.1, 2.8	Patient-centered implementation
3. Telehealth is a priority applied pharmacy competence	Clinical application	2.3	Remote care and telepharmacy readiness
4. Patient-facing digital technologies should be taught as integrated clusters	Practical digital tools	2.4–2.6, partly 2.7	Avoid fragmented teaching
5. Health information systems are essential practice infrastructure	Practice systems	3.3, 3.4	Clinical workflow integration
6. Artificial intelligence is the dominant curricular priority**	Highest-ranked competence	4.2, partly 4.3	Urgent curricular inclusion
7. Precision medicine and public health informatics are relevant but less universal	Advanced/future-oriented areas	4.1, 4.4	Pathway-specific or advanced training
8. The need for pharmacy-specific framework adjustment.	Meta-level critique	Cross-cutting	Pharmacy tailoring of framework
9. Feasibility is shaped by barriers, enablers, and contextual factors	Implementation conditions	Cross-cutting	Readiness and delivery
10. AI is not only content but also a pedagogical disruptor**	Assessment and governance	Cross-cutting	Educational redesign

*Themes 1 and 2 share a literacy-adjacent vocabulary, reflecting different competency domains (DECODE Domain 1 vs. Domain 2) and generating distinct curriculum implications. **Themes 6 and 10 have different curriculum implications, as Theme 6 addresses AI as the dominant curricular priority, and Theme 10 addresses AI as a pedagogical disruptor.

The identified themes were as follows:

1. Foundational digital professionalism is non-negotiable. Ethics, legal/regulatory issues, digital identity, safety, and security were consistently treated as core foundations for any digital health curriculum: *“(…) we all mean that we need a legal and ethical base to approach digital health.” (Workshop 1); “But we actually agreed on 1.1 very fast because we need a solid basis when we talk about digital health.” (Workshop 2).*
2. Digital health literacy is a transversal competence. Participants framed digital health literacy as central to patient communication, equity, counselling adaptation, and safe implementation of digital tools: *“(…) important for the students to understand how to explain in a simple way this digital approach to the population” (Workshop 1); “(…) if you do not have patients that have enough health literacy, then it does not matter how much you provide your counseling good if they cannot understand it. “(Workshop 2).*
3. Telehealth is a priority applied pharmacy competence. Telehealth was seen as increasingly relevant for telepharmacy, rural access, remote consulting, and person-centered medicines-related decision making: *“(…) student is able to make safe and person-centered clinical decisions by using remote consulting skills.” (Workshop 1); “(…) telehealth would be important to have in the curriculum because actually it’s already happening and we assume that it’s going to grow.” (Workshop 2).*
4. Patient-facing digital technologies should be taught as integrated clusters. Digital diagnostics, sensors/wearables, health apps, and digital therapeutics were repeatedly bundled together as overlapping practical areas rather than separate topics: *“We do digital diagnostics using sensors and the results are shown in the health apps. So it’s one point for us as well” (Workshop 1); “(…) clinical trials are quite moving from medicines to medical devices, and now more and more we have combined products.” (Workshop 2).*
5. Health information systems are essential practice infrastructure. Electronic health records and information exchange were prioritized because of their direct relevance to medication review, documentation, and continuity of care: *“(…) a good way would be to produce a medication review report using the available electronic health record data.” (Workshop 1); “(…) electronic health records is important… focuses more on actually using it, and we thought once you use it, you’ll recognize the importance of it (…)” (Workshop 2)*
6. Artificial intelligence is the dominant curricular priority. AI emerged as the strongest agreed topic, but participants emphasized that it should be taught critically, including risks, limitations, validity, and changing applications: *“(…) important for students to understand… that not everything is exactly as AI says, but it’s still a worthy tool to use.” (Workshop 1); “(…) they learn what it is and what it is not and what it is not yet and what it might be — which is very hard to teach but they should realize that it’s a moving target (…)” (Workshop 2).*
7. Precision medicine and public health informatics are relevant but less universal. These were valued, but often positioned as pathway-specific, advanced, or less central than AI, literacy, telehealth, or EHRs: *“Precision medicine, we also think it’s something that could be of highly important in the future.” (Workshop 1); “Precision medicine… It is a hot topic and it’s almost not feasible without digital tools.” (Workshop 2).*
8. The need for pharmacy-specific framework adjustment. Participants perceived overlaps, inconsistencies, passive wording, and a medical bias in the framework, leading them to merge, reframe, or re-sequence competencies: *“(…) we need something more active, so by defining the learning outcomes, we need to be more specific and competencies-based.” (Workshop 1); “(…) it tries to present a series of competencies to a certain profession, but at the same time, It’s somehow it’s too broad. “(Workshop 2).*
9. Feasibility is shaped by barriers, enablers, and contextual factors. Implementation discussions highlighted faculty expertise, curriculum crowding, resources, governance, accreditation, and institutional digital maturity as major determinants of feasibility: *“The education of teachers… Who is going to teach this. The educational of academics that will teach this.” (Workshop 1); “(…)no way, because there’s no money and no people to guide that and no political will to eliminate other stuff” (Workshop 2).*
10. AI is not only content but also a pedagogical disruptor. AI reshaped discussions on assessment, academic integrity, oral examinations, faculty policy, and student anxiety, making it both a curriculum topic and an implementation challenge: *“(…) how do we evaluate their knowledge in this setting, knowing that most of them will do their essays another work assessment. (…) that is a big issue now.” (Workshop 1); “(…) this is probably a matter of how they were educated until they came to your stuff [oral exam]. And we see very much the same thing. Although it’s maybe improving already a little bit, but this mental health stuff is so boring. I mean, get real. And if you do not know anything, what will you tell to the patients? So that’s what we have to tell them, actually.” (Workshop 2).*

Themes 1 to 7 relate primarily to competency selection and curricular content, Theme 8 to framework adaptation, and Themes 9 and 10 to implementation feasibility and educational consequences (Table 2). Theme 1 (Foundational digital professionalism) and Theme 2 (Digital health literacy) are analytically distinct: Theme 1 addresses institutional and regulatory foundations — ethics, legal obligations, digital identity, and security — that apply to the pharmacist as a professional. Theme 2 concerns the communicative and patient-facing dimension of digital literacy — assessing patients’ digital capabilities, adapting counselling, and supporting equitable access to digital tools. While these themes share a literacy-adjacent vocabulary, they reflect different competency domains (DECODE Domain 1 vs. Domain 2) and generate distinct curriculum implications. Theme 6 (AI as dominant curricular priority) and Theme 10 (AI as pedagogical disruptor) are also analytically separable: Theme 6 addresses what AI content should be taught and why it is prioritized; Theme 10 addresses how AI’s pervasiveness reshapes the conditions of teaching, assessment, and institutional governance — making it an environmental factor as well as a content topic.

The following sections present the results of the workshops through this thematic lens.

### 3.2 Competency prioritization

Competency vote counts reflect the prioritization exercise within this purposive workshop sample and should be understood as structured elicitation of expert opinion, not as Delphi-level or population-representative consensus. Across both workshops, eight small groups (four per workshop) independently reviewed and prioritized digital health competencies from the DECODE framework adapted for pharmacy.

Table 3 presents the full competency voting matrix and prioritization regarding direct count of votes and workshoped rationale. Four competencies achieved workshop-level agreement across all eight groups. Artificial intelligence in healthcare (Competency 4.2) was selected by all four groups in each workshop, achieving the maximum score of 8 out of 8. Electronic health records (3.3) similarly achieved selection by all eight groups. Ethics, legal, and professional considerations in digital health (1.1) were directly selected by 7 groups, with one group (WS1) selecting 1.2 (Digital Identity & Security) instead of 1.1, reflecting the view that security, digital identity, and data protection are inseparable from the ethical and legal foundations of digital health practice, and that both competencies should be treated as a unified foundational block. Digital health literacy (2.1) was selected by 7 groups, with universal selection in Workshop 2 and three of four groups in Workshop 1, being framed by all as transversal competence for patient communication, equity, and counselling adaptation.

**Table 3:** Competency prioritization across workshop groups (*N* = 8 groups).

**Competencies**		**WS1** (votes)	**WS2** (votes)	**Total** (/8)	**Key rationale**
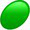 WS-level agreement (7–8/8), 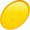 Strong support (5–6/8), 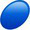 Moderate support (3–4/8), and 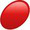 Minimal or no support/deferred (0–2/8)					
4.2	AI in healthcare	4	4	8	Universal priority across all groups. AI described as a “moving target” requiring critical understanding of limitations, biases, and evolving applications.
3.3	Electronic health records (EHRs)	4	4	8	Essential for medication review, e-prescribing, and e-health hygiene. Proposed as experiential placement activity.
1.1	Ethics, legal and professional foundations in digital health	3	4	7	Treated as the non-negotiable foundational layer for any digital health curriculum.
2.1	Digital health literacy	3	4	7	All agreed in WS2; 3/4 in WS1. Framed by all as transversal competence for patient communication, equity, and counselling adaptation.
2.3	Telehealth	1	4	5	Strong support especially in WS2. Telepharmacy growth, remote consulting skills, and person-centered decision-making cited.
2.5	Sensors, wearables and internet of things (IoT)	3	2	5	Bundled with 2.4 and 2.6 in most groups. Validity of data, patient education, and opportunity-focused framing emphasized.
3.4	Health information exchange and personal health records	3	1	4	Consistently paired with 3.3 as a single data management cluster. Data sharing and continuity of care rationale.
2.6	Health applications and digital therapeutics	3	1	4	Linked to DiGA register; combined with 2.4 and 2.5 in practice-oriented bundles. Patient counselling focus.
1.2	Digital identity and security	1	1	2	Selected by 2 groups (one as substitute for 1.1). IT security understanding relevant but absorbed into 1.1 by most.
3.5	Human-centered design in digital health	1	1	2	Cited as a key reason digital solutions fail in practice. Low vote count but conceptually valued.
4.4	Precision medicine	1	1	2	Almost not feasible without digital tools. Positioned as pathway-specific or advanced/CPD content.
4.1	Public health informatics	0	1	1	Enables discovery of related digital competencies. Positioned as elective or advanced.
4.3	LLMs / Computational thinking	1	0	1	Proposed reframing: replace “computational thinking” with dedicated Large Language Models (LLMs) competency.
2.7	Internet-based health interventions	0	0	0	Actively excluded by most groups: “Everything digital is internet these days. Why bother?” Subsumed into broader digital health topics.
2.8	Digital determinants of health	0	0	1	Absorbed into 2.1 Digital Health Literacy or public health modules. Not seen as standalone undergraduate competency.
3.1 3.2	Data governance and data quality	0	0	0	Unanimously deferred to CPD / postgraduate programs. Requires professional maturity and institutional context beyond undergraduate level.

Workshop 1 (WS1): 4 groups, *n* = 14 participants | Workshop 2 (WS2): 4 groups, *n* = 16 participants | Total of 8 groups voting, prioritized by descendent order of votes.

Telehealth (2.3) and sensors, wearables, and the Internet of Things (2.5) each received support from five groups, representing strong but not universal agreement. Health information exchange and personal health records (3.4) and health applications and digital therapeutics (2.6) were each selected by four groups, indicating moderate support. Several competencies received minimal or no support: internet-based health interventions (2.7) was actively excluded by most groups, with one participant arguing, “How would you distribute an app without the internet? Everything digital is internet these days. Why bother?” Data governance competencies (3.1, 3.2) were unanimously deferred to continuing professional development or postgraduate programs.

AI emerged as the highest-ranked competency in this sample, but participants emphasized the need to teach it with nuance. As one participant articulated:

*"They learn what it is and what it is not and what it is not yet and what it might be — which is very hard to teach — but they should realize that it's a moving target, and it's sometimes moving very fast, but there have been AI winters before, and they should realize that." (Workshop 2, Group C)*

A recurring pattern was the bundling of related competencies during group deliberations. Groups consistently combined digital diagnostics, sensors/wearables, and health applications (2.4 + 2.5 + 2.6) into a single teaching unit; merged EHRs with personal health records and data sharing (3.3 + 3.4); and linked digital health literacy with digital determinants of health (2.1 + 2.8). This tendency toward bundling reflected participants’ view that the DECODE framework’s granularity, while useful as an analytic tool, did not reflect how these competencies are encountered or taught in practice.

A notable finding was the proposal to reframe computational thinking (4.3) as a dedicated large language model (LLM) competency. One participant argued:

*"Computational thinking, I think it's totally out. I would replace LLMs. So instead of computational thinking, I think the large language models, they deserve separate attention." (Workshop 1, Group 3)*

The same participant elaborated on the interconnection between LLMs and communication skills:

*"You really cannot teach communication in pharmacy and counseling without also teaching based on information navigation. And when you go into that, then you're going to run into things like LLMs and Dr. Google and various sources of information which we should be able to teach our students to use." (Workshop 1, Group 3)*

Beyond the DECODE competencies, participants identified additional competency areas that they considered absent or inadequately represented: innovation and entrepreneurship in digital health, LLMs as a standalone competency distinct from generic AI, communication in digital contexts, information navigation and critical appraisal of digital sources, digital ethics as a domain separate from legal and regulatory considerations, and sex, gender, and diversity considerations in digital health.

Taken together, the prioritization exercise did not simply generate a ranked list of DECODE items. It also revealed a process of pharmacy-specific reinterpretation, in which participants selectively endorsed, merged, reframed, or downgraded competencies according to perceived relevance, teachability, and curricular fit. Overall, competency prioritization mapped most strongly onto Themes 1–6, especially the centrality of foundational professionalism, digital health literacy, health information systems, and AI.

### 3.3 Module outlines

Across both workshops, eight module outlines were co-designed by the participant groups, covering the highest-priority competencies. Table 4 summarizes the modules, their target learners, key learning outcomes, teaching methods, and assessment approaches. The modules spanned a broad range of digital health topics and collectively addressed all four DECODE domains.

**Table 4:** Summary of co-designed module outlines across both workshops.

**Module focus**	**Key learning outcomes (Bloom’s level)**	**Teaching methods**	**Assessment**
EHR (3.3)	Understand data sources; Apply: medication review using EHR; Evaluate: judge gaps in EHRs	Exploratory examples; experiential learning with real EHR data during placement	Practical placement assessment; peer transfer exercise
Health Apps (2.6)	Name definitions/specifications; List reliable databases; Match apps to target groups; Apply: solve problems using apps	Blended learning; classroom instruction; problem-based learning	Problem-solving: justify app selection for clinical scenario
LLMs (4.3 reframed)	Understand LLM principles; Evaluate LLM versions; Apply LLMs in pharmacy scenarios	Lectures; PBL; case-based learning; direct LLM use	Case-based application scenarios
Telehealth (2.3)	Describe when/how to use telehealth; Discuss benefits/limitations; Solve DRPs via remote consulting; Person-centered decisions	Simulated remote consulting	Performance-based remote consulting assessment
Digital health literacy (2.1)	Understand: describe theory, measurement; Apply: construct literacy profiles; Analyze: investigate/appraise impact on outcomes	Seminars; case studies; patient scenarios	Case study performance; seminar participation
Digital health literacy + AI (2.1, 4.2)	Assess patient digital literacy; Identify tools; Adapt communication; Define AI/ML; Check AI tool validity; Apply AI in pharmacy	Not specified	Not specified
AI in healthcare (4.2)	Understand: AI/ML types and data processes; Analyze: scope and limitations; Evaluate: appropriate use in healthcare	Lectures; prompt engineering; case-based learning	Poster/infographic; tool selection debate; case-based group discussion
Ethics/Legal/Regulatory (1.1)	Understand ethical, legal, regulatory standards; Understand patient/carer/professional perspectives	Case studies; films; peer-generated ethical challenges; interprofessional groups	Observation; peer assessment; reflection on peer-created cases

The electronic health records module proposed an innovative experiential learning approach: during the practical placement year, one student would conduct a medication review using available EHR data and transfer the findings to a peer, who would then interview the actual patient to identify what the EHR captured and what it missed. This exercise was designed to reveal the strengths and limitations of EHRs for clinical decision-making through direct experience.

The health applications module emphasized self-directed, problem-based learning, with students tasked to identify appropriate health applications from reliable source databases (such as the German DiGA register) rather than using pre-selected examples. The LLM module represented a reframing of computational thinking (DECODE Competency 4.3) and included prompt engineering as both a teaching method and a skill.

Telehealth focused on remote consulting skills, with learning outcomes explicitly framed around person-centered clinical decision-making. Digital health literacy was structured around three overarching learning goals with outcomes progressing from understanding through application to analysis, using seminars and patient case studies.

In the digital health literacy modules, participants repeatedly emphasized that literacy should not be conceptualized as passive awareness. Instead, it should involve assessment of patients’ literacy levels, adaptation of counselling, and in some cases follow-up or re-evaluation of whether digital solutions are actually being used effectively.

Two groups addressed AI in healthcare: one combined it with digital health literacy, incorporating patient literacy assessment and AI tool evaluation; the other focused specifically on AI, proposing learning outcomes at the understand, analyze, and evaluate levels with corresponding assessments including poster creation, prompt engineering exercises, and case-based discussions. The ethics/legal/regulatory module proposed an interprofessional case-based approach involving films, peer-generated ethical challenges, and observation-based assessment - drawing on an existing model at one institution where ethical case discussions already involved seven to eight health professions.

Across all modules, several pedagogical patterns were consistent. Problem-based learning and case-based learning were the most frequently proposed teaching methods. Bloom’s taxonomy was explicitly referenced in structuring learning outcomes across understanding, application, analysis, and evaluation levels. Blended learning was proposed for foundational knowledge delivery to avoid unnecessary in-person instruction for factual content. Prompt engineering emerged as a novel, AI-specific active learning and assessment method. Experiential assessment during placement years was proposed for applied competencies such as EHR use and telehealth consulting. These patterns are important because they suggest that participants did not see digital health education as requiring entirely new pedagogical paradigms. Instead, they tended to adapt familiar educational formats already used in pharmacy education, such as PBL, case discussions, experiential placements, and seminars, to new digital content. This may increase practical feasibility.

### 3.4 Framework integration and curricular sequencing

Across both workshops, participants consistently distinguished between competencies appropriate for different stages of pharmacy education. A three-tier curricular structure emerged from the framework integration discussions.

Foundational (Years 1–2). Ethics, legal, and regulatory foundations (1.1, 1.2), digital health literacy (2.1), and basic AI/LLM orientation were positioned as foundational. Participants argued that early LLM introduction was essential to address academic integrity from the outset: “In the very beginning, they need to get to know the LLM, their advantages and also restrictions.” Basic awareness of major technology categories (sensors, telehealth, health applications) was recommended at a conceptual level during this phase.

Mid-curriculum (Years 3–4). Clinical skills-oriented competencies — telehealth and remote consulting (2.3), sensors, wearables, and health applications (2.5, 2.6), and electronic health records (3.3) — were positioned in the mid-curriculum, where students would encounter them in clinical contexts. One participant articulated the sequencing logic: “They need to know the basics and then they know the diagnostics of the diseases and then we just transfer it into the digital diagnostics.” This stage was also where participants most often favored integration with placements, practical classes, or scenario-based teaching rather than a purely theoretical approach. The mid-curriculum thus appears to be the preferred zone for moving from awareness to application.

Advanced (Year 5/Placement/CPD). Full-depth AI in healthcare (4.2), precision medicine (4.4), and data governance (3.1, 3.2) were positioned at the advanced or postgraduate level. LLMs at the advanced application level (prompt engineering for specific pharmacy use cases) and internet-based health interventions (2.7), if addressed at all, were also placed here. Importantly, “advanced” did not always mean “late undergraduate.” In some groups, advanced digital topics were considered more appropriate for placement-based teaching, CPD, or specialized postgraduate pathways, especially when institutional infrastructure or faculty expertise were limited.

A key structural proposal, articulated by one Workshop 2 group, was to divide competencies into three categories: mandatory for all undergraduate students, optional or pathway-specific (allowing specialization in areas such as precision medicine or digital counseling), and postgraduate (for advanced data governance or informatics). This three-category model was seen as more realistic than a single undifferentiated competency list.

Overlap analysis revealed considerable redundancy across DECODE competencies as perceived by participants. The most frequently proposed mergers included: 2.4 + 2.5 + 2.6 (digital diagnostics, sensors, health applications) as a single teaching unit; 3.3 + 3.4 (EHRs and personal health records); 2.1 + 2.8 (digital health literacy and digital determinants); and 4.2 + 4.3 (AI and LLMs, with the latter reframed). One group went further, proposing that all of 2.3–2.7 be combined into a single domain, arguing that the individual competencies were too granular for practical teaching.

The debate on whether digital health should be a standalone module or integrated across the curriculum was a prominent theme. One participant advocated for a dedicated module:

*"The advantage of having a separate model is that you can really quantify how much it is. Because if it's disseminated, depending on the teaching staff, it will be more or less. And the level will be also different." (Workshop 1, Group 2)*

Another participant countered that integration was more appropriate:

*"I think of digital health, security — those sort of things — also that we need to teach in parallel, but it's integrated in what we are doing. So it's not that we can make just one subject that is focusing on this. This should be integrated in the entire program, starting from year one." (Workshop 1, Group 4)*

The agreed view was that a hybrid approach might be optimal: a dedicated foundational module to establish core concepts and vocabulary, complemented by integration of applied digital health competencies within existing clinical and pharmaceutical care courses throughout the curriculum. This hybrid model also appears to be the most feasible one. It addresses participants’ concern that fully distributed content can become inconsistent and invisible, while avoiding the curricular overload associated with trying to place all digital health content into a single standalone unit.

### 3.5 Feasibility: barriers, enablers, and contextual factors

Participants identified a comprehensive set of factors affecting the implementation of digital health curricula in pharmacy education. Using an inductive-deductive analysis, feasibility-related qualitative data were coded into barriers, enablers, and contextual factors. Table 5 presents the main implementation themes.

**Table 5:** Feasibility-related themes affecting digital health curriculum implementation.

Category	Theme	Illustrative content
Barriers	Curriculum overload and lack of teaching time	Dense curricula; reluctance to remove existing content
	Faculty capacity and expertise gaps	Uncertainty over who can teach advanced digital health
	Limited institutional resources	Lack of time, staff, infrastructure, funding
	Slow curriculum governance	Long approval cycles; national harmonization constraints
	Institutional resistance or inconsistency around AI	Some institutions prohibit AI; others normalize it
	Assessment disruption	Shift toward oral/open assessment due to AI
	Student anxiety and acceptability concerns	Oral exams perceived as more stressful
Enablers	Strong agreement on urgency and relevance	Broad agreement that digital health is now necessary
	Existing partial curricular footholds	Public health, digital pharmacy, digital skills modules already exist
	Active traditions	PBL, case-based and experiential methods already familiar
	Interprofessional collaboration	Pooling expertise across health disciplines
	Elective/pilot pathways	Pragmatic entry route before formal embedding
	Accreditation/regulatory pressure	External mandates can accelerate implementation
	Student and institutional AI adoption	Existing AI use forces engagement
Contextual factors	UG vs. CPD/postgraduate distinction	Some competencies seen as core, others as advanced
	Cross-country and sector variability	Relevance depends on practice setting and national system
	Institutional digital maturity	Universities differ in readiness, guidance, and culture
	Need for pharmacy-specific adaptation	DECODE perceived as medically oriented and too granular

Curriculum overload was the most frequently cited barrier. As one participant noted, digital health inclusion would require curricular realism, particularly in science-heavy pharmacy programs “We really need to be reasonable and not to jump from the, like in Germany, a lot of pharmaceutical chemistry into internet-based.” Several participants stressed that feasibility was limited not by lack of relevance, but by lack of curricular space and by the absence of political willingness to remove or reduce existing material.

Faculty capacity and expertise emerged as the primary human resource barrier, with participants acknowledging that the question of “who is going to teach this” remained unresolved. One participant described the likely initial state of implementation:

*"At the beginning it will be a bit patchy. It won't be consistently the same quality across all the different things. But there are different people who have already taken it up within pharmacy and within related health science areas. One of the easiest ways, from a sort of skills point of view, but most difficult from a logistic point of view, is to do it together with some of the other health science people. Because then you're pooling your talents from the different groups." (Workshop 2)*

Institutional inertia was particularly evident in attitudes toward AI. One participant observed:

*"I have the impression in my university there are still teams and groups who say no use of AI, we don't want that. And I think that's totally wrong and from yesterday. And so we're really behind the curve. And I think we need people coming in who tell us how you can do it constructively and proactively." (Workshop 2)*

Another participant responded even more bluntly to institutions maintaining AI prohibitions: “You are in denial.”

Among enablers, accreditation body mandates were identified as the single most powerful mechanism for system-wide change:

*"If the accreditation body comes in and says, okay, you've revised the criteria, here we are, and this is the timeline, then everybody has to do it. There's no option." (Workshop 2)*

The Norwegian model was highlighted as a concrete example: the EU directive on digital competencies had already been translated into national regulations requiring university students to possess digital competencies, e-health skills, and digital security awareness. Portugal’s approach of providing institutional access to major AI tools for university students was cited as an enabler of proactive engagement with AI in education. However, participants also identified more incremental enablers. These included starting with elective or pilot offerings, embedding digital health into existing modules, and leveraging established pedagogical traditions such as PBL, seminars, interprofessional case teaching, and experiential placements. In this sense, feasibility was frequently negotiated through adaptation rather than wholesale curricular replacement.

Assessment adaptation emerged as both a barrier and a driver of change. Several participants had shifted to oral examinations in response to AI-generated student work. More broadly, participants described a transition away from conventional written assignments toward assessment formats that privilege explanation, justification, discussion, and live performance. This reinforces the idea that digital health implementation is inseparable from a broader redesign of assessment in the AI era. However, participants also raised concerns about the emotional toll of this shift on students, noting that oral examinations had caused anxiety and distress (25). The need to balance assessment integrity with student wellbeing was recognized as a significant implementation consideration.

### 3.6 DECODE framework

Although DECODE served as a useful starting point for structuring discussions, participants - particularly in Workshop 2 - raised substantive concerns about its fitness for purpose in the pharmacy context.

The most prominent comment concerned the framework’s origins in medical education. Participants did not reject the framework though. Rather, they used it as a scaffolding device and then engaged in substantial reinterpretation. The thematic coding showed four recurring adaptation moves: (1) prioritizing only a subset of competencies as essential; (2) bundling overlapping competencies into teachable clusters; (3) reframing selected competencies, especially computational thinking into LLM-related content; and (4) redistributing some competencies to CPD, placements, or pathway-specific training. Specific concerns included the use of passive, recognition-level language in competency descriptions. The verb “to recognize” was identified as insufficiently active for competency-based pharmacy education, with participants arguing that learning outcomes should use action-oriented verbs that specify demonstrable skills. As one participant articulated: “We need something more active, so by defining the learning outcomes, we need to be more specific and competencies-based.” Furthermore, participants proposed separating ethics from legal/regulatory content, as these were viewed as “very broad fields and they both deserve separate sort of attention.” The philosophical distinction between what is ethical and what is regulated was emphasized: “There are lots of stupid regulations,” implying that ethical reasoning requires independent critical thinking that cannot be reduced to regulatory compliance.

Participants also identified a limitation-focused bias in the DECODE framework’s language (emphasis on “recognizing scope and limitations”) and called for an opportunity-focused reframing that better reflects the expanding role of pharmacists as active users and innovators of digital health technologies.

A further criticism concerned the framework’s internal consistency. Participants observed that similar competency formulations were unevenly distributed across topics. For example, some competencies emphasized scope and limitations, while others foregrounded use or management, which made the structure feel arbitrary and harder to translate into coherent pharmacy teaching units. This contributed to the repeated calls for consolidation, simplification, and pharmacy-specific redesign.

### 3.7 Integrative thematic synthesis

When the competency coding and the feasibility analysis are considered together, four higher-order results emerge.

First, participants consistently favored a pharmacy curriculum based on a small number of core, high-value competencies rather than exhaustive coverage of all digital health domains. AI, EHRs, digital health literacy, telehealth, and ethical/security foundations formed the clearest undergraduate core. These were seen as broadly applicable across pharmacy sectors and sufficiently foundational to justify mandatory inclusion.

Second, participants preferred integrated, practice-oriented competence clusters over highly granular frameworks. The most stable clusters involved digital literacy plus digital determinants; telehealth plus digital diagnostics, wearables, apps, and digital therapeutics; EHRs plus information exchange/personal health records; and AI plus LLM-related critical use. This clustering reflects how pharmacists are likely to encounter digital technologies in practice rather than how they appear in taxonomic frameworks.

Third, feasibility was constrained less by resistance to the idea of digital health than by implementation readiness. Participants generally agreed on the value and urgency of digital health education. The major obstacles were not conceptual but operational: lack of faculty expertise, limited curriculum space, slow governance, and uncertainty about assessment in the AI era.

Fourth, AI occupied a uniquely disruptive position. It was simultaneously the highest-priority competence, the strongest driver of curriculum reform, and the factor most clearly exposing institutional inconsistency. In practice, AI forced discussions not only about what should be taught, but also about how students should be assessed, what counts as valid learning, and whether institutions were adapting fast enough to digital change.

While the workshop design did not include systematic sub-group comparisons, several patterns were observed informally. Academic pharmacists tended to frame competency priorities in terms of curricular integration and assessment feasibility, while community and clinical pharmacists more frequently foregrounded patient-facing digital tools (health applications, wearables, and telepharmacy) and workflow-level EHR use. It should be noted, however, that many participants operated across both settings - a characteristic of the PCNE network that mitigates sharp academic-practitioner divides and ensures that curricular priorities reflect practice realities directly. Workshop 2 participants tended to raise more systematic concerns about the DECODE framework’s medical education orientation and proposed more extensive restructuring than Workshop 1.

As the study prioritized the identification of convergent competency priorities to develop a transferable curriculum blueprint, no systematic country-level differences were identifiable in the transcripts. Nonetheless, participants from countries with more advanced national digital health regulation cited specific accreditation mandates and policy frameworks as implementation enablers - contextual factors that participants from countries at earlier stages of digital health policy development did not raise. These observations are exploratory and are not based on formal sub-group analysis; future studies with larger, more structured samples would be required to investigate such contextual variation systematically.

## 4 Discussion

This study presents an early-stage multinational, co-designed digital health curriculum blueprint, specifically tailored to pharmacy education in Europe. Through two structured expert workshops engaging 30 participants from 13 European countries, four core competency pillars emerged with workshop-level agreement: artificial intelligence in healthcare, ethics and legal/professional considerations in digital health, electronic health records, and digital health literacy. These four pillars provide a clear starting point for pharmacy curriculum developers seeking to prioritize digital health content within already crowded programs. Importantly, these high-ranking competencies were not prioritized only because participants viewed them as conceptually important. Rather, they were repeatedly described as foundational, cross-cutting, and immediately relevant to pharmacy education and practice. In particular, ethics and security, digital health literacy, EHRs, and AI were framed as the minimum core that all pharmacy graduates should acquire, regardless of their eventual sector of practice. This suggests that participants were not advocating for maximal inclusion of all available digital health topics, but for a smaller and more defensible undergraduate core.

Beyond competency prioritization, the study generated eight co-designed module outlines that translate abstract competencies into actionable curriculum components with defined learning outcomes, teaching methods, and assessment strategies. The three-tier sequencing framework (foundational, mid-curriculum, advanced) and the structural distinction between mandatory core, pathway-specific, and postgraduate content offer a flexible architecture that can be adapted across diverse pharmacy education systems. A strong emphasis on active, application-level learning outcomes emerged, with participants consistently pushing beyond the DECODE framework’s “recognize” language toward action-oriented competency statements. The critical appraisal of the DECODE framework itself is therefore a significant finding, highlighting the need for pharmacy-specific adaptations of medical education competency frameworks. The proposed modules further revealed a strong preference for authentic and performance-based assessment, particularly in areas where digital competence was seen as inseparable from judgment, communication, and professional reasoning.

A further principal finding was the strong resistance to fragmented curricular design. Participants consistently bundled related competencies into practice-oriented clusters - for example, telehealth, digital diagnostics, wearables, apps, and digital therapeutics; or digital health literacy and digital determinants of health. This indicates that, from a pharmacy education perspective, digital health may be better approached through integrated competence clusters than through highly granular frameworks.

Finally, AI occupied a qualitatively different position from the other topics. It was not discussed merely as another curricular area, but as both a content priority and a curricular disruptor. Participants framed AI as unavoidable for future pharmacy practice, academic work, and assessment design, making it simultaneously a learning domain, a practice reality, and a driver of institutional change.

### 4.1 Alignment and coherence

The competency priorities generated in this study are notable not only for their alignment with the FIP global survey gaps (1), but for what they reveal about how pharmacy educators conceptualize the scope of digital health education itself. The FIP report documented that clinical reasoning and evidence-based digital medicine, alongside cybersecurity and patient empowerment, were the least-taught digital health topics - and the same domains emerged here as high-priority areas requiring integration. Crucially, however, participants were not calling for the wholesale addition of digital health as a new thematic block. Rather, they consistently distinguished between what all graduates must be able to do (a defensible mandatory core), what can be addressed at awareness level, and what is more appropriate for postgraduate or CPD contexts. This granular differentiation represents a more operationally useful contribution than a simple competency list: it provides curriculum developers with a principled basis for prioritization in an environment where programs are already crowded and faculty time is constrained.

The DECODE framework (6), developed through an international Delphi process for medical education, provided a valuable structural starting point. However, our participants’ critical engagement with DECODE, particularly with regard to its passive language, possible clinical bias and limitation-focused framing, suggests that direct transposition to pharmacy is insufficient. In an interdisciplinary context such as this digital health curriculum, it should always be approached as an orientation and ground for working a real curriculum that prioritizes the needs of each workgroup (in this case the pharmacy workforce). This finding extends the work of Mantel-Teeuwisse et al. (5) who documented pharmacy programs’ limited preparedness and responsiveness to digital health, by providing a concrete adaptation pathway. The emphasis on action-oriented learning outcomes and pharmacy-specific contextualization reported here offers a bridge between the generic DECODE taxonomy and the practical needs of pharmacy educators. In this sense, DECODE appears best understood not as a ready-made curricular architecture for pharmacy, but as a starting taxonomy that requires translation, consolidation, and local adaptation. This gives opportunity for a proper translation to other professional settings and ensures ownership. The apparent tension on using DECODE as a central scaffold while critiquing its fitness for pharmacy is intentional and analytically productive. DECODE was not adopted as a ready-made pharmacy curriculum but as one of the most rigorous, internationally validated digital health competency taxonomy currently available, providing a principled starting point for structured elicitation. The value of using DECODE lay precisely in its ability to provoke pharmacy-specific reinterpretation: by exposing participants to a structured competency list developed for the medical settings, the workshops generated systematic evidence about what pharmacy educators would retain, merge, reframe, or reject. This productive friction between framework and context is consistent with the principles of the ADDIE Analysis phase, which is designed to surface misalignments between existing frameworks and local needs. DECODE is therefore best understood in this study as a generative scaffold rather than a prescriptive template - a role that the study’s findings both demonstrate and validate.

The barriers and enablers identified in this study also resonate with broader literature on digital health workforce development and curriculum reform. The recurring concerns around fragmented provision, insufficient faculty capacity, and absence of standardized digital health teaching are consistent with wider observations across European health professions education. Our participants’ repeated identification of accreditation bodies and regulatory mandates as key levers of change also fits with the broader literature suggesting that curricular reform in health professions is rarely scaled through isolated institutional initiative alone. Rather, it is often accelerated through external standards, competency requirements, and quality assurance mechanisms. The role of faculty development deserves particular emphasis here. One of the clearest findings from our feasibility analysis was that digital health implementation was repeatedly framed as contingent on the education of educators themselves. This aligns closely with the FIP-reported gap of insufficient expert capacity and suggests that staff development may be as important as student curriculum reform.

The unanimous prioritization of AI across all eight workshop groups is relevant not merely as a vote count, but as a signal of where pharmacy educators perceive the most urgent capability gap. The proposal to distinguish large language models as a discrete competency area separate from generic AI reflects participants’ awareness that LLMs are already shaping student behavior - through routine use of tools such as ChatGPT or similar - in ways that existing competency frameworks have not yet addressed. This represents a structural gap: students are using tools that require skills in prompt engineering, critical evaluation of AI outputs, and responsible academic use, while curricula remain silent on these capabilities. More broadly, the variation participants described between institutions that have normalized AI use and those still focused on prohibition or plagiarism detection points to a growing divide in institutional digital maturity that will likely become an increasingly important explanatory variable in future studies of digital curriculum reform and educational equity.

### 4.2 Implications for practice

The three-tier sequencing architecture proposed in this study addresses a practical tension that is rarely resolved in digital health curriculum literature: how to introduce new content into programs that are already full. By distinguishing between foundational modules (suitable for immediate implementation), mid-curriculum integration, and advanced or pathway-specific content, the framework accommodates the curriculum revision timelines of 5–7 years cited by participants as a structural constraint. This is analytically important because it reframes digital health integration not as a one-time wholesale revision - an approach participants viewed as unrealistic - but as a staged process that allows institutions to act now on the highest-priority competencies while planning more systematic integration over time. The distinction between mandatory core and pathway-specific content further accommodates the diversity of pharmacy education systems across Europe and beyond. The principle of interprofessional co-teaching similarly addresses resource constraints without requiring institutional investment in new specialist faculty - one of the most frequently cited barriers. Taken together, these design choices reflect an implementation-conscious logic that distinguishes this framework from competency lists that stop at content specification without addressing how institutions might realistically enact them.

The findings also suggest that curriculum developers should resist the temptation to build overly fragmented digital health curricula. Participants consistently preferred integrated competence clusters, especially for patient-facing technologies. From a practical standpoint, this means that teaching telehealth, digital diagnostics, wearables, apps, and digital therapeutics as interconnected areas may be more educationally coherent and more feasible than treating them as fully separate modules. The recurrence of curriculum crowding, faculty capacity, and institutional digital maturity as barriers across all groups suggests that digital health integration will, in many institutions, require a negotiated rather than comprehensive approach - one that prioritizes high-agreed competencies first and builds capacity incrementally.

Accreditation bodies emerge from this study as key change agents. The shared view that accreditation mandates are the most frequently cited lever for system-wide change in this workshop sample suggests that advocacy directed at accreditation organizations may be more productive than institution-by-institution curriculum reform. The Norwegian example of translating EU digital competency directives into national educational requirements provides a concrete model for policy transfer. However, systemic change through accreditation bodies is slow and politically dependent. Participants therefore also identified more incremental, institution-level routes that do not require regulatory change. These included starting with electives or pilot offerings, embedding digital health into existing modules, and building on established pedagogical traditions such as PBL, seminars, interprofessional case teaching, and experiential placements. This suggests that implementation may often proceed more realistically through adaptation and staged embedding than through wholesale curricular replacement.

Faculty development programs in digital health represent an urgent priority. The repeated identification of faculty expertise gaps as the primary barrier, combined with the observation that faculty often lag behind students in AI adoption, underscores the need for parallel investment in educator training alongside curriculum development. The “patchy” quality acknowledged as inevitable in early implementation makes a compelling case for communities of practice among digital health pharmacy educators. In practical terms, educator development should not be limited to technical digital knowledge. It should also include curriculum design, case development, assessment redesign, ethical reasoning in digital contexts, and facilitation of active learning around AI and digital tools.

LLMs require particularly urgent curricular attention. The finding that students are already using these tools routinely - while institutional responses range from prohibition to mandatory integration - creates a gap that formal education must fill. The proposed reframing of computational thinking as an LLM-specific competency, with dedicated attention to prompt engineering, output evaluation, and ethical use, represents a timely and actionable recommendation. The implications for assessment are equally important. Participants’ preference for authentic and performance-based assessments indicates that digital health education may require a shift away from formats that privilege recall alone and toward formats that assess explanation, justification, professional judgment, and live interaction. However, our findings also caution that this redesign must account for student anxiety and the pedagogical acceptability of more oral or performance-based assessment modes.

### 4.3 Theory of change and global health workforce impact

Building on WHO’s guidance on evidence-informed theories of change (21), we articulated an explicit ToC for the proposed curriculum. The qualitative, stakeholder-driven ADDIE process aligns with ToC principles by specifying the mechanisms through which a co-designed, competency-based curriculum is expected to contribute to a more digitally capable pharmacy workforce. In the Analysis phase, the ToC assumes that systematically identifying competence gaps and stakeholder priorities generates a shared problem definition. In the Design and Development phases, the co-creation of learning outcomes is posited to strengthen educator ownership, increasing the likelihood that the curriculum will be implemented with fidelity.

**Figure 1:**
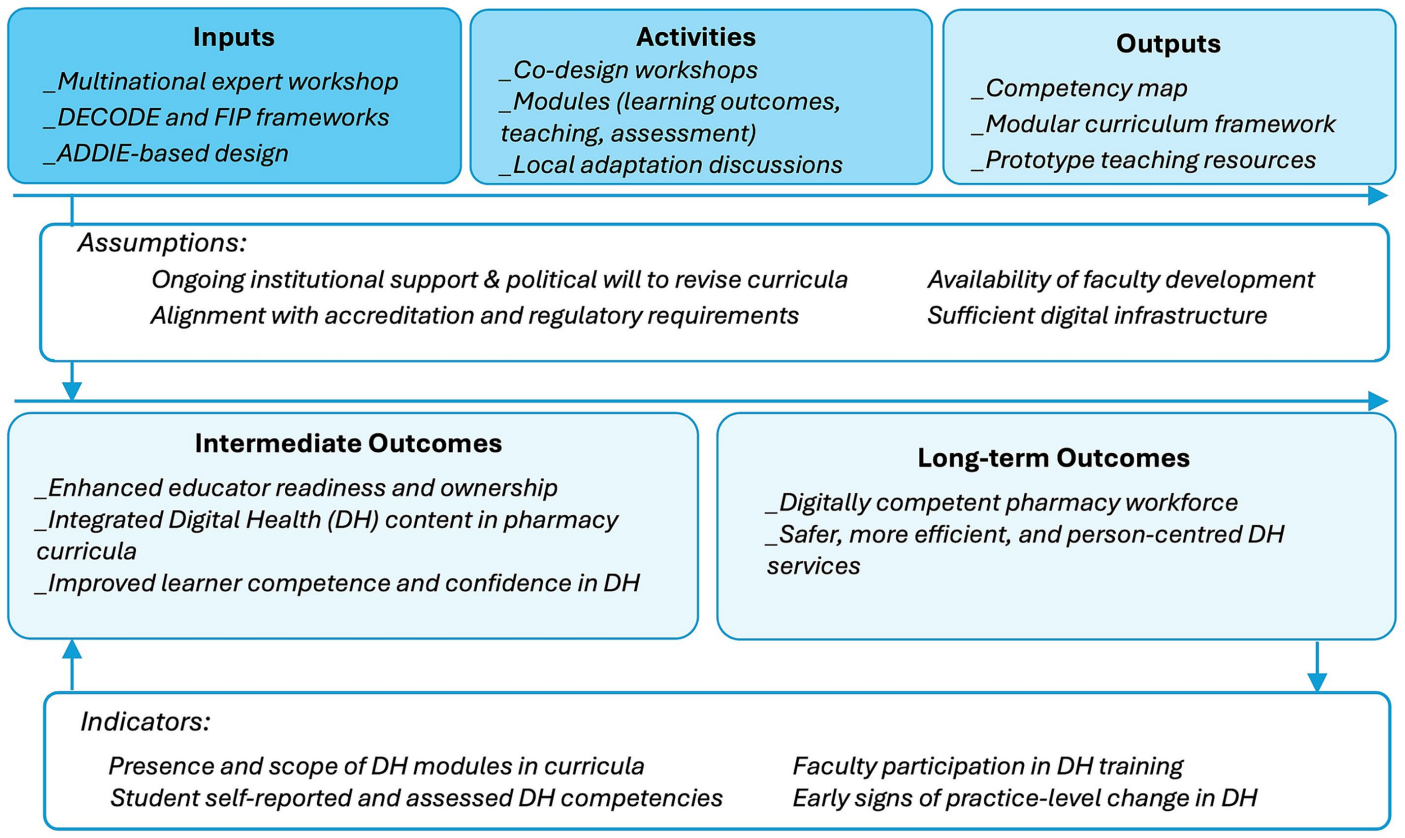
Theory of Change for the modular digital health curriculum in pharmacy education, showing the pathway from inputs and activities to outputs, intermediate outcomes, and long-term workforce impacts, together with key assumptions and example monitoring indicators.

As illustrated in Figure 1, inputs - including stakeholder expertise, digital infrastructure, and institutional support - feed into co-design and pilot activities to generate concrete outputs such as competence maps and prototype modules. These are expected to produce enhanced educator readiness and ownership, integrated digital health content in pharmacy curricula, and improved learner competence and confidence in digital health. The ToC further anticipates both primary and secondary long-term impacts. Positive primary effects include the institutionalization of digital health in pharmacy education, contributing to a digitally competent workforce and safer pharmaceutical care. The model also proactively accounts for potential negative secondary effects, notably student anxiety arising from the shift toward oral or performance-based assessments in response to AI-related academic integrity concerns.

The ToC explicitly links theoretical assumptions to the empirical risks identified during the workshops, with a particular focus on implementation and feasibility. A primary assumption is the availability of faculty with sufficient digital health competence; however, participants identified “faculty capacity and expertise” as a major barrier, asking pointedly: “Who is going to teach this?” A further critical assumption is institutional support and political willingness, which is countered by the risks of “institutional inertia” and “curriculum crowding.” Experts noted there is often “no political will to eliminate other stuff” to accommodate new digital content, and some institutions were described as being “in denial” regarding the pace of digital change, particularly concerning AI.

To mitigate these risks, the ToC recognizes accreditation body mandates as a critical enabler; as one participant observed, when a mandate is issued, “then everybody has to do it. There’s no option.”

Future evaluation should therefore use this ToC as a monitoring framework, tracking the following indicators:

- Institutionalization: the presence and scope of dedicated digital health modules in partner curricula.
- Capacity building: faculty participation in professional development programs to bridge the identified expertise gap.
- Learner impact: documented (self-reported and assessed) student digital competence gains, weighed against potential negative secondary effects such as anxiety and distress arising from the transition to oral or performance-based assessments.
- Practice change: documented signals of change in digital practice.

Findings from these evaluation activities should feed back into the model, acknowledging that the curriculum is a dynamic artefact. This iterative process ensures that the ToC serves as an ongoing reference point as the project moves from the Design phase into the Implementation and Evaluation phases of the ADDIE cycle.

From the Theory of Change lens, the co-design methodology provided a structured opportunity for a workshop-level agreement-building around a curriculum that addresses prioritized digital health needs in pharmacy education while remaining adaptable to diverse implementation contexts. The process brought together researchers and practitioners, facilitating shared understanding and co-ownership of the innovation and equipping participants with both a framework and practical tools to operationalize it within their own institutions. This flexibility is crucial in a landscape characterized by heterogeneous organizational cultures, regulatory environments, career structures, and workforce pressures; for some settings, the curriculum may be most feasible as a stand-alone mandatory course, whereas in others it may be better integrated as smaller elective modules or embedded components of existing courses. Such contextual tailoring is fully consistent with a ToC perspective that emphasizes local pathways to shared outcomes rather than a single uniform implementation route.

Beyond pharmacy, the interdisciplinary framework and modular structure proposed here are inherently transferable. The same co-design logic could be applied to other health professions and interprofessional programs, allowing them to harmonize digital health learning around shared competency clusters while addressing their own specific needs and priorities. This is particularly relevant given the severe health workforce challenges currently documented across Europe, including acute shortages, retention problems, and high levels of burnout. The WHO European Region’s Framework for action on the health and care workforce 2023–2030 (26) calls for strategies that improve working conditions, strengthen digital skills, and support data-driven workforce planning. A competency-based digital health curriculum, such as the one developed in this study, can serve as a practical vector of change, creating conditions for safer, more efficient use of digital tools, and thereby contributing to the broader goal of optimizing human resources for health, including the pharmacy workforce.

In this sense, the present study contributes both as a call to action and as a concrete response. It offers actors involved in pharmacy education - academics, professional bodies, students, and regulators - a set of tools and a framework for implementing digital health education in varied European contexts. By validating a competency-based, instructional design-driven methodology, it provides a replicable approach for other programs seeking to integrate digital health. The proposed curriculum aims to contribute to the global framework of competencies required for effective pharmaceutical workforce performance (27) and aligns with ongoing efforts to strengthen the health workforce in the WHO European Region (26).

Our findings also resonate with the most recent WHO Europe report on artificial intelligence in health care across European Union Member States (28). That report provides the first region-wide snapshot of AI adoption, highlighting uneven readiness, substantial variation in regulatory and governance arrangements, and persistent gaps in workforce skills and training for AI-enabled care. The strong prioritization of AI and, uniquely, of LLM in our workshops directly mirrors these system-level concerns, suggesting that pharmacy educators are acutely aware of emerging AI-related capability gaps. By specifying AI- and LLM-related learning outcomes, teaching strategies, and assessment approaches, the curriculum proposed in this study offers a concrete educational response to the report’s call for systematic capacity-building in AI literacy, critical appraisal, and responsible use of AI tools in clinical and educational practice. In doing so, it positions pharmacy education as an important lever for operationalizing the recommendations of the WHO Europe AI report at the level of day-to-day teaching and learning.

### 4.4 Strengths and limitations

Several notable strengths should be highlighted. The multinational expert panel, comprising 30 participants from 13 countries, provides a breadth of perspective that institution-specific or single-country studies cannot achieve. The use of a structured co-design methodology grounded in validated frameworks (DECODE, ADDIE, Theory of Change) enhances methodological rigor and transferability. Real-time qualitative data capture through audio recording and transcription of all plenary discussions yielded rich, detailed accounts of participant reasoning. The inclusion of both academic and community pharmacy perspectives ensured that the proposed modules reflect diverse practice settings, and the workshop format - combining structured activities with open discussion - balanced efficiency with depth of engagement. A further strength is that the study did not stop at identifying priorities; it also documented how participants reasoned about bundling, sequencing, feasibility, adaptation, and implementation, allowing the results to speak not only to “what should be taught” but also to “how this might realistically be done.”

Several steps were taken to strengthen the trustworthiness of the analysis. The study used data triangulation across two workshops and eight small-group outputs. Competency vote counts were interpreted alongside plenary qualitative discussions, allowing comparison between what groups selected and how they justified those selections. The analytic process combined framework-informed coding with openness to emergent themes, particularly in the feasibility analysis. The use of AI tools was explicitly bounded to an assistive role, with all final coding and interpretation undertaken by the research team. The workshop format and facilitation approach may have influenced which issues were emphasized in plenary discussion, and the research team’s interest in digital health curriculum development may have shaped interpretation; to mitigate this, the analysis remained grounded in transcript material and privileged recurring themes supported across multiple groups rather than isolated comments.

Participation was voluntary and self-selected via open workshop registration. No formal exclusion criteria were applied. The total number of conference attendees eligible to register was not formally recorded; therefore, an exact participation rate cannot be reported. This is acknowledged as a limitation of conference-embedded recruitment.

The sample was exclusively European and drawn from PCNE membership and related networks. Although the study was grounded in FIP guidelines and included participants with global FIP affiliations, the geographic scope limits direct transferability to non-European contexts. A further limitation concerns conference-based selection bias. Participants were drawn from attendees of the PCNE Working Symposium, a network explicitly associated with pharmaceutical care research and innovation. This likely overrepresents pharmacists who are already engaged with educational innovation and digital health, relative to the broader pharmacy academic community. It follows that the proposed competency priorities and curriculum architecture may reflect the perspectives of early adopters rather than the mainstream of pharmacy education. Purposive sampling through a professional network, while appropriate for an expert co-design study, introduces the risk of systematic alignment between participant values and the study’s goals. At the same time, these participants can be seen as potential “champions” well positioned to introduce the digital health curriculum within their own institutions, an analytically useful feature that does not substitute for broader representativeness.

Critically, undergraduate pharmacy students - the intended beneficiaries of any resulting curriculum - were not represented in either workshop. While the participant pool was notably heterogeneous, spanning diverse professional roles, occupational positions, and national contexts, the absence of learner perspectives remains a meaningful gap. Patients, public representatives, employers, and national regulatory authorities, although present as professional identities within some participants’ broader roles, were not formally included as distinct stakeholder groups in the workshop design. Future validation phases should therefore actively seek to incorporate structured learner and patient/public input, particularly through surveys or consultations with student cohorts and patient communities, to complement and test the expert-derived framework produced here.

The findings represent primarily the Analysis and Design phases of ADDIE; the Development, Implementation, and Evaluation phases constitute future work. The curriculum framework presented here should therefore be understood as a co-designed blueprint for intervention rather than a fully tested program. Finally, the prominence of AI and LLM-related themes likely reflects their current immediacy and visibility, and may have amplified their relative emphasis compared with other digital health domains that are equally important but less conspicuously disruptive in day-to-day educational practice.

### 4.5 Future directions

The immediate next step is the development phase: producing prototype module packages for the highest-priority competencies, particularly AI, EHRs, ethics/legal-professional foundations, and digital health literacy. Pilot implementation in partner institutions across Europe will test the feasibility and acceptability of the proposed modules in authentic pharmacy education and continuing professional development settings. Evaluation should include learner outcomes, educator experiences, implementation barriers, and local adaptation needs. Future evaluation should also pay close attention to whether the integrated competence-cluster approach suggested by participants produces better educational coherence and feasibility than a more fragmented competency-by-competency design.

Broader validation through a Delphi study or large-scale survey, incorporating pharmacy educators, students, practicing pharmacists, patients, and regulatory stakeholders from beyond Europe, would strengthen the external validity of the competency priorities and curriculum framework. Integration with FIP Development Goal 20^2^ monitoring would align the curriculum with global pharmacy development indicators. The establishment of communities of practice for digital health pharmacy educators, potentially leveraging networks such as PCNE, could support knowledge exchange, resource sharing, and collaborative quality improvement.

More broadly, our findings indicate that future framework development should not focus only on competency content. It should also explicitly address sequencing, modularity, educator preparation, assessment implications, and the distinction between undergraduate core, pathway-specific content, and postgraduate or CPD learning. In this sense, the present study supports moving from static lists of digital competencies toward more implementation-conscious curricular models.

## 5 Conclusion

This study identifies a pharmacy-relevant core for digital health education: AI in healthcare, ethics/legal-professional foundations, electronic health records, and digital health literacy. It also shows that pharmacy educators prefer integrated, practice-oriented competence clusters over highly granular frameworks, and favor a staged curriculum that distinguishes between core undergraduate, pathway-specific, and postgraduate/CPD content.

The main challenge is not whether digital health should be included in pharmacy education, but how it can be implemented under real institutional conditions. Feasibility was shaped by faculty expertise, curriculum crowding, governance, accreditation, and institutional digital maturity, with AI standing out as both the strongest curricular priority and the most frequently cited lever for system-wide change in this workshop sample. Theory of Change together with an instructional competencies-based approach offers a structured logic model linking design inputs to expected workforce outcomes, with assumptions and indicators to be tested in future evaluation phases. Overall, this study provides a pharmacy-specific, implementation-conscious blueprint to guide the next phase of curriculum development, piloting, and evaluation.

## Data Availability

The datasets presented in this article are not readily available because in line with GDPR principles and the irreversible anonymization commitments described in the Ethics section, raw workshop transcripts and audio recordings cannot be shared publicly, as doing so would risk re-identification of participants in a small, expert community. The article was prepared with as much detail as possible, without compromising participant confidentiality or the risk of re-identification. This is covered by: (1) the anonymized competency voting matrices and prioritization (Table 2), and (2) the DECODE competency pool worksheet used in Activity 1 (Supplementary material). The study was pre-registered on the Open Science Framework (doi: 10.17605/OSF.IO/S54J8). Requests to access the datasets should be directed to melanie.maia@ihmt.unl.pt.

## Footnotes

1 Open Science Framework pre-registration. Team, M. (2026, February 16). Modular Curriculum on Digital Health for the Pharmacy Context. doi:10.17605/OSF.IO/S54J8.

2 FIP Development Goal 20, https://developmentgoals.fip.org/dg20/. Accessed in 30 April 2026.
