# Supplementary material for: Designing a modular digital health curriculum for pharmacy education: a stakeholder-driven expert workshop study
Source: Front Public Health. 2026 Jul 29;14:1888486. doi: 10.3389/fpubh.2026.1888486 (PMC13462222; doi:10.3389/fpubh.2026.1888486)
Supplement: Supplementary file 1 [file Data_sheet_1.pdf]

DECODE Framework

A) Aim: modular curriculum digital health for pharmacy context

B) Target (included profiles): **pharmacy science professional/student**

Proficiency Levels: ***Foundational*** ***Advanced*** Digital Health: DECODE mandatory learning outcomes

D) Competencies Matrix

Competencies		Description	Learning Outcomes *"At the end of the learning pathway, students will..."*
**Domain 1: Professionalism in Digital Health**			
	1.1: Professionalism, ethical, legal, and regulatory considerations in digital health	To demonstrate adherence to digital health professional, ethical, legal, and regulatory standards when handling health information.	
	1.2: Digital identity, safety, and security	To maintain appropriate professional digital identity, apply concepts of digital intelligence to healthcare, and recognize their own critical role in cyber-risks and -threats.	
**Domain 2: Patient, consumer, and population digital health**			
	2.1: Digital health literacy	To recognize the importance of digital health literacy of the population and associated health inequalities.	
	2.2: Personal health records	To recognize the role of personal health records in patient-centered care.	
	2.3: Telehealth	To recognize the scope, capabilities, and limitations of telehealth and demonstrates remote consulting skills.	
	2.4: Digital diagnostics	To recognize the scope, abilities, and limitations of digital diagnostics and demonstrates their efficient use relevant to the field of practice.	
	2.5: Sensors, wearables, and IoT	To identify the scope of wearables and internet of things (IoT) in patient care and recognize their limitations.	
	2.6: Health apps and digital therapeutics	To demonstrate knowledge of health apps and digital therapeutics.	
	2.7: Internet-based health interventions	To describe different designs and areas of clinical applications of internet-based health interventions.	
	2.8 Digital Determinants of Hea	To describe various digital determinants of health and recognize their impact on digital health equity.	

Competencies	Description	Learning Outcomes<br>*"At the end of the learning pathway, students will…"*
**Domain 3: Health information systems**		
3.1: Data governance and data management	To manage health data efficiently in accordance with respective healthcare data governance standards.	
3.2: Foundation and principles of health information systems	To demonstrate secure and efficient operation and management of health data using health information systems.	
3.3: Electronic health records	To demonstrate appropriate use of electronic health records, e-prescribing, and other EHR-related clinical software relevant for the area of practice and geographic region.	
3.4: Health information exchange	To demonstrate the ability to operate electronic health information exchange in a manner that optimizes safe and effective patient- and population-centered care.	
3.5: Human-centered design in digital health	To apply human-centered design and system thinking when creating or evaluating digital health solutions for patients and health professionals.	
**Domain 4: Health Data Science**		
4.1: Public health informatics	To describe the application of informatics in the public health field.	
4.2: Artificial intelligence in healthcare	To identify the scope of data analytics, AI, and machine learning in healthcare, as well as describe how solutions based on these may be used to achieve the best patient, population, and health system outcomes.	
4.3: Computational thinking in healthcare	To apply the principles of computational thinking to healthcare.	
4.4 Precision Medicine	To describe the concepts of precision medicine, its intersection with digital health, and its applications and relevance for public health and health of an individual patient.	

Other competencies not mentioned before:
